# Colonization with multidrug-resistant organisms is associated with in increased mortality in liver transplant candidates

**DOI:** 10.1371/journal.pone.0245091

**Published:** 2021-01-22

**Authors:** Philip G. Ferstl, Natalie Filmann, Eva-Maria Heilgenthal, Andreas A. Schnitzbauer, Wolf O. Bechstein, Volkhard A. J. Kempf, David Villinger, Tilman G. Schultze, Michael Hogardt, Christoph Stephan, Haitham Mutlak, Nina Weiler, Marcus M. Mücke, Jonel Trebicka, Stefan Zeuzem, Oliver Waidmann, Martin-Walter Welker

**Affiliations:** 1 Department for Internal Medicine I / Gastroenterology and Hepatology, University Hospital, Goethe University, Frankfurt am Main, Germany; 2 Institute of Biostatistics and Mathematical Modeling, Goethe University, Frankfurt am Main, Frankfurt, Germany; 3 Department of General and Visceral Surgery, University Hospital, Goethe University, Frankfurt am Main, Germany; 4 Institute for Medical Microbiology and Infection Control, University Hospital, Goethe University, Frankfurt am Main, Germany; 5 University Center for Infectious Diseases (UCI), University Hospital Frankfurt, Goethe University, Frankfurt am Main, Germany; 6 University Center of Competence for Infection Control of the State of Hesse, Frankfurt Main, Germany; 7 Department for Internal Medicine II / Infectious Diseases, University Hospital, Goethe University, Frankfurt am Main, Germany; 8 Department of Anesthesiology, Intensive Care Medicine and Pain Therapy, University Hospital, Goethe University, Frankfurt am Main, Germany; Medizinische Fakultat der RWTH Aachen, GERMANY

## Abstract

**Objectives:**

Rising prevalence of multidrug-resistant organisms (MDRO) is a major health problem in patients with liver cirrhosis. The impact of MDRO colonization in liver transplantation (LT) candidates and recipients on mortality has not been determined in detail.

**Methods:**

Patients consecutively evaluated and listed for LT in a tertiary German liver transplant center from 2008 to 2018 underwent screening for MDRO colonization including methicillin-resistant Staphylococcus aureus (MRSA), multidrug-resistant gram-negative bacteria (MDRGN), and vancomycin-resistant enterococci (VRE). MDRO colonization and infection status were obtained at LT evaluation, planned and unplanned hospitalization, three months upon graft allocation, or at last follow-up on the waiting list.

**Results:**

In total, 351 patients were listed for LT, of whom 164 (47%) underwent LT after a median of 249 (range 0–1662) days. Incidence of MDRO colonization increased during waiting time for LT, and MRDO colonization was associated with increased mortality on the waiting list (HR = 2.57, p<0.0001. One patients was colonized with a carbapenem-resistant strain at listing, 9 patients acquired carbapenem-resistant gram-negative bacteria (CRGN) on the waiting list, and 4 more after LT. In total, 10 of these 14 patients died.

**Conclusions:**

Colonization with MDRO is associated with increased mortality on the waiting list, but not in short-term follow-up after LT. Moreover, colonization with CRGN seems associated with high mortality in liver transplant candidates and recipients.

## Introduction

Liver transplantation (LT) is an established treatment in patients with acute liver failure and advanced liver disease with and without hepatocellular carcinoma [1, 2]. Bacterial infections are a major cause of short-term mortality after LT, and unresolved infections are considered a contraindication against liver transplantation [3]. Moreover, infections are a major trigger of acute-on-chronic liver failure in patients both with compensated and decompensated cirrhosis [4–6].

Rising prevalence of multidrug-resistant organisms (MDRO) and especially colonization with multidrug-resistant gram-negative bacteria (MDRGN) is considered one of the most crucial and yet unresolved problems in public healthcare [7, 8]. In a study of 475 liver graft recipients from Asia, MDRGN were identified as the dominant pathogens in liver transplant recipients [9]. Moreover, infections with MDRO in patients with cirrhosis have been associated with increased mortality [10, 11] and liver transplant recipients have a substantial risk for infections with MDRO [12, 13]. Furthermore, infections with MDRGN are a serious complication after LT [13, 14]. The situation is less clear for colonization with MDRO, especially in LT candidates or recipients with vancomycin-resistant enterococci (VRE) colonization. So far, data are scarce with respect to current prevalence of MDRO colonization in LT candidates and even more the impact of MDRO colonization on the mortality prior and after LT.

In Germany, organ shortage results in a high model of end stage liver disease (MELD) score at LT and a prolonged waiting time between listing for LT and graft allocation [15, 16]. It is accepted that an uncontrolled infection regardless of MDRO status remains a contraindication against LT in common. However, there is uncertainty about the prospects of MDRO-colonized patients awaiting LT. The aim of the current study was to determine the prevalence of MDRO colonization at our center at the time point of listing, and the incidence of new MDRO colonization during the waiting time for LT. Moreover, we addressed whether MDRO colonization is associated with higher mortality prior and after LT.

## Patients and methods

### Study design

This retrospective study was conducted at a German tertiary liver care unit in patients with indication for LT. The standard LT evaluation protocol included a screening protocol for MDRO colonization comprising of nasopharyngeal/throat, rectal, and optional cutaneous smear samples/swabs. Following the recommendations of the German Commission for Hospital Hygiene and Infection Prevention (KRINKO), the local hospital’s infection control strategy requires MDRO screening in case of hospitalization and/or admission to the intermediate and intensive care unit [17]. Inclusion criteria contained evaluation and formal registration for LT and age of at least 18 years. Study approval was obtained by the local Ethics Committee for Medical Research of the Medical Faculty, University Hospital, Goethe University, Frankfurt am Main, in accordance with the 1975 Declaration of Helsinki prior to research (file number 268/13). Informed consent was obtained upon study inclusion, and the database was pseudo-anonymized. Patients who had been listed for LT, but died before informed consent could be obtained, were included into the analysis in accordance with the Ethics Committee vote. Screening for MDRO colonization had been repeated at planned and emergency hospitalization events including time point of LT. Moreover, laboratory and clinical data were collected and analyzed retrospectively including infectious complications. Follow-up period was at least one year after evaluation for LT, or at least three months in patients who received a liver graft. Data acquisition was closed by July 2019, and thus six patients without LT were followed up for less than one year (286, 319, 321, 354, 357 and 361 days). Fatal outcome was defined as primary end point, and patients who did not reach this end point were censored from the last day of follow-up. Fatal outcome due to severe infection, tumor progression, and other causes were defined as secondary endpoints. Survival status of patients who were lost to follow-up was surveyed by telephone interview, in correspondence with hospitals of referral, or via public registration office inquiry. Data from individual patients may have been reported previously with respect to different topics [18–20].

### Definition of MDRO, colonization, invasive detection, infection, and severe infection

In the current study, MDRO were defined as MDRGN, VRE and methicillin-resistant Staphylococcus aureus (MRSA), as defined earlier [21]. In particular, MDRGN are defined as *Enterobacterales* with extended spectrum beta-lactamase (ESBL) phenotype as well as *Enterobacterales*, *Pseudomonas aeruginosa*, and *Acinetobacter baumannii* resistant against Piperacillin, any 3rd/4th generation cephalosporin, and fluoroquinolones [22]. CRGN are a subgroup of MDRGN that, beside ESBL phenotype (Enterobacterales) or resistance against piperacillin, ceftazidime and fluoroquinolones (*P*. *aeruginosa*), carry additional resistance to carbapenems [23]. Colonization with MDRO was defined as detection of MDRO in rectal, pharyngeal/throat, or cutaneous screening samples. Importantly, multiple different MDRGN strains may have been detected within single patients. Moreover, all MDRO strains isolated from ascites, blood, urine, bronchial or pleural secretion, bile, pus, wounds or surgical sites, on medical devices or indwelling catheters were defined as invasive MDRO detections. Diagnosis of infection was based on the combination of invasive detection and according clinical parameters, *i*.*e*. fever or laboratory signs of infection. The quick sequential organ failure assessment (qSOFA) score was applied to stratify severity of infection as recommended [24].

### Detection of MDRO and molecular resistance analysis

For MDRGN screening CHROMagar^™^ ESBL plates (Mast Diagnostica, Paris, France) were used allowing the detection of drug-resistent *Enterobacterales*, *P*. *aeruginosa*, and *Acinetobacter* spp. Screening for VRE and MRSA colonization was performed using VRE selective agar (bioMérieux, Nuertingen, Germany) and Briliance MRSA 2 agar (Oxoid, Wesel, Germany), respectively. All recovered isolates were tested for their respective resistance profile. All laboratory procedures were performed under quality-controlled standards (laboratory accreditation according to ISO 15189:2007 standards; certificate number D-ML-13102-01-00, valid through January 25, 2021), as described earlier [25].

### Statistical analysis

#### Baseline parameters

Statistical analyses were conducted with BiAS software (v11.06; Epsilon-Verlag, Darmstadt, Germany) and R (version 4.0.2, R Core Team (2020), Vienna, Austria, packages kmi and survival). Categorical variables were described as frequencies and percentages. Continuous variables were presented as medians or means, with ranges or quartiles, as appropriate. The Wilcoxon-Mann-Whitney-U-test was used for comparisons of quantitative and ordinal variables at baseline or at LT. All tests were two-sided and p-values ≤0.05 were considered statistically significant. Calculating the probability of undergoing LT, LT itself was considered as endpoint, and de-listing and death were defined as competing event.

#### Time-dependent endpoints and competing risks

Importantly, clinical endpoints were assessed depending on MDRO acquisition, which is a time-dependent variable. Regarding time-to-event analyses, the subsequent outcomes were analyzed with regard to the following details. i) Death on the waiting list as the event of interest up to one year after MDRO screening, considering de-listing and LT as competing events. ii) Death due to severe infection as the event of interest, with death due to other causes than severe infection being defined as additional competing risk. iii) We also analyzed the incidence of MDRO colonization on the waiting list by registering all new MDRO detections as a time-dependent variable. Finally, cumulative incidence of new MDRO colonization was calculated for patients who were MDRO-negative at study inclusion. Hereby death, LT, and de-listing were regarded as competing events. Results were depicted as log-hazard ratios (log-HR). Independent risk factors for events of interests were calculated using uni- and multivariate Cox regression (death), and a proportional sub-distribution hazards’ regression model (all other end points). Hereby, MDRO colonization was included as time-dependent factor.

#### Endpoints in LT recipients with a “clock-reset” at LT

Since LT is a curative approach in cirrhosis, a different hazard for the abovementioned endpoints and competing risks was to be expected upon LT. In this particular analysis, LT was defined as time point zero, and we assessed the cumulative incidences of MDRO colonization up to 3 months after LT as a time-dependent variable, as described above. Hereby death was regarded as competing event. We also conducted a time-to-event analysis of death and death due to severe infection up to 3 months after LT, where for the latter case death due to other causes than severe infection was defined as competing risk.

## Results

### Study population

In the present study, 351 patients were included between December 2008 and November 2018. Alcohol- (109/351, 31%) and HCV-associated (103/351, 29.3%) liver disease were the leading causes for cirrhosis (S1 Table). Following listing, 164/351 (46.7%) patients with a median (min., max.) MELD score of 16 (7, 40) points underwent LT after a median (min., max.) of 249 (0, 1662) days (Table 1). At study inclusion, MELD score was significantly lower in non-MDRO patients (median 15, IQR 10–21) than in MDRO-positive patients (median 18, IQR 14–25, p = 0.0026). However, at LT MELD was not different between non-MDRO patients (median 19, IQR 15–24) and MDRO-positive patients (median 20, IQR 16–25, p>0.2).

**Table 1:** Clinical data at time point of study inclusion and LT.

	At listing (n = 351)	At LT (n = 164)
**Biometric characteristics**		
Male sex	234 (66.7%)	109 (66.5%)
Age (years)	53.6 ± 10.1	53.8 ± 10.8
Days in study (Mean ± SEM)	0	284 ± 258
**Liver scores**		
MELD	16.7 ± 7.6	19.9 ± 6.8
Child A	83 (23.6%)	3 (1.8%)
Child B	121 (34.5%)	47 (28.7%)
Child C	147 (41.9%)	114 (69.5%)
**Laboratory parameters**		
CRP (mg/dl)	1.5 ± 2.5	4.8 ± 4.3
Creatinine (mg/dl)	1.2 ± 0.8	1.3 ± 0.8
Urea (mg/dl)	43.5 ± 32.6	50 ± 27.2
Bilirubin (mg/dl)	5.8 ± 7.9	5.7 ± 5.4
Albumin (mg/dl)	3.5 ± 1.8	2.4 ± 0.6
ALT (U/l)	157.5 ± 541.3	1089.5 ± 998.5
AP (U/l)	177.2 ± 163.7	111.2 ± 74
gGT (U/l)	163.6 ± 239.7	118.8 ± 100
INR	1.5 ± 0.6	1.8 ± 0.7
TSH (mU/l)	2.3 ± 1.6	2.7 ± 3.2
Leukocytes/nl	6.1 ± 3.1	9.5 ± 5.9
Hemoglobine (g/dl)	11.5 ± 2.4	10.1 ± 1.5
Thrombocytes/nl	119.6 ± 84.1	104.3 ± 58.8
AFP (ng/ml)	29.4 ± 113.8	3.9 ± 4.2
**History of decompensation**		
HE	95 (27.1%)	59 (36%)
SBP	41 (11.7%)	20 (12.2%)
Variceal bleeding	75 (21.4%)	24 (14.6%)
HRS	22 (6.3%)	21 (12.8%)
PVT	17 (4.8%)	9 (5.5%)
HCC	103 (29.3%)	61 (37.2%)*

For continuous variables, mean value ± standard deviation are given. Abbreviations: CRP, C-reactive protein. ALT, alanine transaminase. AP, alkaline phosphatase. gGT, gamma-glutamyltransferase. INR, international normalized ratio. TSH, thyroid-stimulating hormone. AFP, alpha-fetoprotein. HE, hepatic encephalopathy. SBP, spontaneous bacterial peritonitis. HRS, hepatorenal syndrome. PVT, portal vein thrombosis. HCC, hepatocellular carcinoma.

### MDRO prevalence and incidence in LT candidates and recipients

At listing, 70/351 (19.9%) patients were colonized with MDRO, of whom 31/351 accounted for MDRGN (8.8%; thereof 1 CRGN, 0.3%), 13/351 (3.7%) for MRSA and 35/351 (10.0%) for VRE. Among 351 patients initially screened for any MDRO, 261/351 (74.3%) repeatedly underwent further MDRO screening on the waiting list (S2 Table). The cumulative 1-year MDRO incidence on the waiting list was 33.3%, comprising patients with MDRGN (17.5%; thereof CRGN 2.3%), MRSA (5.0%), and VRE (22.3. At LT, 60/164 patients were MDRO-positive (MDRGN, n = 30 [thereof CRGN, n = 2]; MRSA, n = 5; VRE, n = 37), resulting in a prevalence of 36.6% for any MDRO, 18.8% for MDRGN (thereof 1.2% CRGN), 3.0% for MRSA and 22.6% for VRE. The cumulative MDRO incidence in LT recipients at 3-months follow-up rose to 47.3% (MDRGN 32.4% [thereof 1.9% CRGN], MRSA 5.7%, VRE 37). Until death or last follow-up, 73/164 LT recipients were tested positive for any MDRO (MDRGN, n = 50 [thereof CRGN, n = 4]; MRSA, n = 9; VRE, n = 57). Among 163 patients who remained on the waiting list after one year, 28 (17.7%) carried MDRO, with 18 (11%) being positive for MDRGN (thereof 3 CRGN, 1.8%), 4 (2.5%) for MRSA and 14 (8.6%) for VRE. Throughout the entire cohort, 173/351 patients had been tested MDRO-positive in total (MDRGN, n = 89 [thereof CRGN, n = 14]; MRSA, n = 19; VRE, n = 123; Table 2, S2–S6 Tables).

**Table 2:** Colonization and infection status, analysis of MDRO prevalence at listing, MDRO cumulative incidence during the course of the study, and clinical outcome depending on MDRO status in 351 patients on the waiting list and 164 patients receiving LT.

	MDRO	MDRGN	Thereof CRGN	MRSA	VRE
**Waiting list (n = 351)**					
Prevalence at listing	70 (19.9%)	31 (8.8%)	1 (0.3%)	13 (3.7%)	35 (10%)
One-year cumulative incidence	33.3%	17.5%	2.3%	5%	22.3%
Invasive MDRO detections on waiting list	124	44	18	4	76
MDRO-positive fatalities	89	43	9	12	60
Death (HR)*	2.57 (p < 0.0001)	1.86 (p = 0.0003)	2.09 (p = 0.0416)	1.94 (p = 0.0217)	2.63 (p < 0.0001)
Death due to severe infection (HR)*	2.07 (p < 0.0001)	1.59 (p = 0.0072)	1.5 (p > 0.2)	1.60 (p = 0.10)	2.1 (p < 0.0001)
**LT patients (n = 164)**					
MDRO-positive patients at LT	60	30	2	5	37
Prevalence at LT	36.6%	18.8%	1.2%	3.0%	22.6%
Invasive detections until LT	38	14	2	2	22
**LT patients at 3-months follow-up (n = 141)**					
MDRO-positive patients	73	50	4	9	57
Three-months cumulative incidence	47.3%	32.4%	1.9%	5.7%	37.4%
Invasive MDRO detections after LT	76	34	6	0	42
MDRO-positive fatalities	14	6	1	1	11
Death (HR)^$^	1.53 (p > 0.2)	0.72 (p > 0.2)	2.97 (p > 0.2)	0.64 (p > 0.2)	1.58 (p > 0.2)
Death due to severe infection (HR)*	2.54 (p = 0.17)	0.6 (p > 0.2)	8.44 (p = 0.06)	n.a.	3.14 (p = 0.10)
**Entire cohort**					
MDRO-positive Patients	173	89	14	19	123

Patients undergoing LT were followed up until death or were censored 90 days after LT. Of note, different MDRO may have been found within individuals. Furthermore, multiple invasive MDRO detections may have been made within single patients. MDRO definition includes MDRGN, MRSA, VRE. CRGN are a subgroup of MDRGN that are resistant against carbapenems beside ESBL phenotype (*Enterobacterales*) or resistance against piperacillin, ceftazidim and fluoroquinolones (*P*. *aeruginosa*).

* = Competing risk model,

^$^ = Cox proportional hazard model.

### MDRO-associated infections in LT candidates and recipients

Altogether, 69 patients with MDRO colonization (29 patients with MDRGN [thereof 8 CRGN]; 1 MRSA; and 46 VRE) who were colonized, also developed infection with the same strain during the course of the study. Among 60 patients tested positive for any MDRO from listing until LT, 38 MDRO-associated infections were detected (MDRGN, n = 14 [thereof CRGN, n = 2]; MRSA, n = 2; VRE, n = 22). The majority of MDRO-associated infections was observed in 26 graft recipients with 76 invasive MDRO detections (MDRGN, n = 34 detections [thereof CRGN, n = 6]; MRSA, n = 0; VRE, n = 42). Among these, 21/26 patients were colonized with the same strain before (MDRGN, n = 11 patients [thereof CRGN, n = 3]; VRE, n = 10). Overall, 41/164 LT recipients showed invasive detection of any MDRO strain before and after LT (18/41 patients with MDRGN-associated infection [thereof 4/41 patients with CRGN-associated infection]; 2/41 patients with MRSA-associated infection; 28/41 patients with VRE-associated infection; Table 2). Throughout the entire cohort, invasive evidence of MDRGN was found in 38/89 patients, MRSA in 2/19 patients, and VRE in 57/123 patients (S3, S4, S5 and S6 Tables). Infection caused by VRE with additional resistance to teicoplanin, linezolid, and/or tigecycline was found in 8 patients (S5 and S6 Tables). Among patients carrying MDRGN, *E*. *coli* was the predominating pathogen, followed by *K*. *pneumoniae* (S7 Table).

### Clinical course in association with MDRO status including mortality analysis

**Fig 1:**
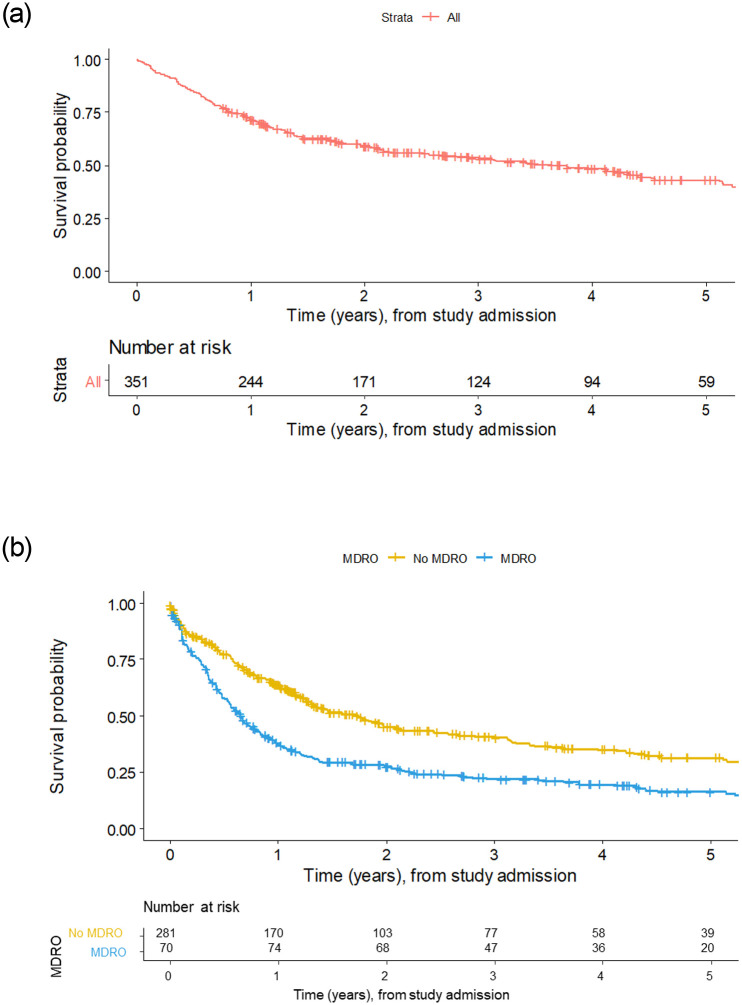
Kaplan-Meier survival plots of all patients included in the current study screening (t = 0) up to loss-to-follow-up, regardless of LT status or de-listing. (A) Kaplan-Meier survival plot of all patients, with number at risk, throughout the study. (B) Patients without MDRO and with patients positive for any of MDRGN, MRSA or VRE. Since MDRO is analyzed as a time-dependent variable, additional patients with newly detected MDRO can appear over time. Therefore, patients at risk are higher after one year in the MDRO group, since these patients had newly acquired MDRO.

**Fig 2:**
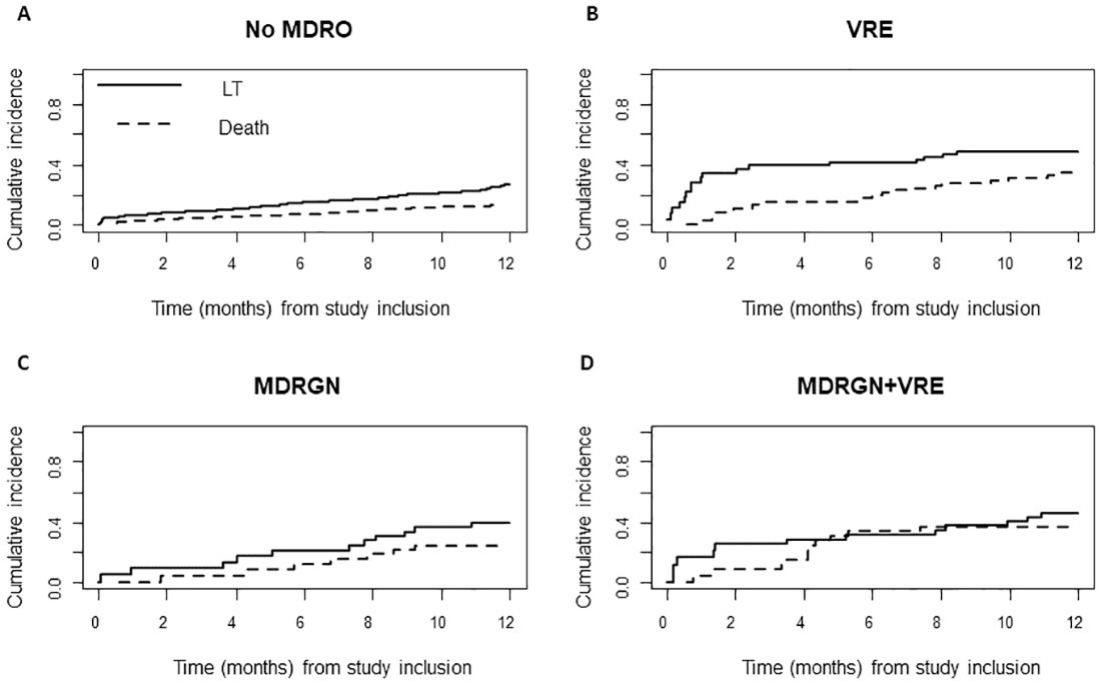
Head-to-head comparison of cumulative incidence functions of survival and LT in patients without MDRO colonization (A), VRE (B), MDRGN (C), and MDRGN+VRE (D).

Among 351 patients, 101 died on the waiting list (79.8% one-year survival rate), and evidence of MDRO was associated with increased risk for death (Fig 1, Table 2). The competing risk analysis showed a higher mortality in patients with MDRO colonization compared to non-colonized patients (HR = 2.57, p<0.0001; Table 2). Furthermore, in patients with MDRO, increased waitlist mortality was observed, and lethal severe infection occurred significantly more often (Fig 2, Table 2). Importantly, in patients receiving a liver graft, MDRO in general were not associated with changes in clinical outcome after LT. However, in patients positive for CRGN (HR = 8.44, p = 0.06) and VRE (HR = 3.14, p = 0.10) a tendency towards lethal infectious complications was observed. Among 74 MDRO-positive patients who had undergone LT, 14 died within 3 months (MDRGN, n = 6 [thereof CRGN, n = 1]; MRSA, n = 1; VRE, n = 11). Among the patients who remained on the waitlist, 89 patients carrying MDRO died (MDRGN, n = 43 [thereof CRGN, n = 9]; MRSA, n = 12; VRE, n = 60). Moreover, mortality was high in patients with CRGN positivity. In detail, 10/14 patients carrying CRGN died during the study, of whom 1/14 had previously undergone LT, while 9/14 died on the waiting list. Uni- and multivariate analysis of potential confounders of MDRO colonization or survival showed that ICU, hepatic encephalopathy, MELD score, recent hospitalization, and recent antibiotic exposure were independent risk factors for death (univariate analysis). In the multivariate analysis, MDRO status, MELD score and recent hospitalization proved to be predictors of death on the waiting list (Table 3).

**Table 3:** Univariate and multivariate analysis for patients on the waiting list regarding overall survival (above) and MDRO acquisition (below).

Univariate analysis—Overall survival*	
Number of hospitalizations during last 12 months	HR = 1.08, p = 0.016
ICU	HR = 2.22, p<0.0001
HE	HR = 1.49, p<0.0001
MELD score	HR = 1.05, p<0.0001
Hospitalization during last 90 days	HR = 1.83, p = 0.0001
Recent antibiotics exposure (30 days)	HR = 1.68, p = 0.0003
**Multivariate analysis—overall survial**^$^	
Any known MDRO	HR = 2.06, p<0.0001
MELD score	HR = 1.04, p<0.0001
Hospitalization during last 90 days	HR = 1.44, p = 0.0221

The following baseline characteristics were regarded: Gender, variceal bleeding, ascites, HE = hepatic encephalopathy, dialysis, ICU = intensive care unit, antibiotic prophylaxis, recent antibiotics exposure (last 30 days), admission to nursing home (no patients dwelling at nursing home), MELD score at listing, number of hospitalizations during the last 12 months, hospitalization during the last 90 days.

* = Univariate model with overall survival up to 12 months on the waiting list,

^$^ = Multivariate model with MRE status (time-dependent).

Among all 164 patients undergoing LT, 23 died within 3 months (90-days survival rate 85.9%). Thereof, 11 died from severe infection, and 12 died from other causes. Within the entire cohort, 146/351 patients died, with 41/146 fatalities from severe infection, 13/146 from tumor progression, and 68/146 from other non-infectious causes. In 24/146 patients, cause of death could not be determined; these fatalities had been reported to us through a public registration office inquiry.

## Conclusions

In the current study, we determined the prevalence of MDRO colonization at the time point of listing for LT, and furthermore the incidence of new MDRO acquisition in initially not colonized patients. Our study demonstrates that MDRO colonization is common in LT candidates and therefore an important medical issue. Moreover, the cumulative incidence of MDRO increased considerably after listing until LT or last follow-up. While a survival benefit in patients undergoing LT is obvious in both in MDRO-positive and negative patients, our study showed that colonization with MDRO was associated with increased mortality in patients on the waiting list.

A detailed analysis of MDRO species showed that mortality was associated with all MDRO subtypes, while lethal infectious complications were mainly associated with VRE. Rising VRE incidence has been observed in LT candidates during the past 15 years [26, 27]. It is not yet conclusively determined whether VRE colonization is a surrogate marker reflecting sicker patients receiving repeated antibiotic exposure that leads to intestinal dysbiosis, or whether VRE colonization has a pathophysiological influence on the clinical course of patients [28]. Indeed, a meta-analysis showed that VRE colonization may prompt infection in solid organ recipients, and amongst these especially in liver graft recipients [27]. A study reporting one-year mortality rates as high as 60% in VRE carriers within a tertiary liver center in the United States did not discriminate between LT candidates and recipients, hence patients at high risk for death due to VRE were not unambiguously identified within this pooled cohort [29]. In our study, detection of VRE was associated with increased risk for death in LT candidates, but not recipients. Therefore, the data from our center support the approach that LT should swiftly be intended in VRE-positive patients.

The second important finding of our study was a noninferior clinical outcome in MDRO-colonized patients who underwent LT, compared to non-MDRO LT recipients (Table 2). In this regard, the correlation between MDRO colonization and MDRO-associated infection must be addressed. Especially in patients with MDRGN positivity, the progression risk from colonization to infection has been well described [12, 13, 30–32]. Since LT is a curative approach in cirrhotic patients, we assume that this also translates into a lesser impact of MDRO-associated lethal complications. However, the situation of CRGN colonization must be addressed separately, because detection of CRGN seems to be associated with deleterious outcome in LT candidates. This is in concordance with other studies indicating an unfavorable course in patients with CRGN colonization [13, 25]. The data of our study do not conclusively clarify whether CRGN colonization may be considered a contraindication to LT. It seems reasonable to consider the given resistance pattern in CRGN-colonized LT candidates with respect to potential rescue antibacterial therapies like ceftazidim/avibactam, cefiderocol, meropenem/varbobactam in the decision process. The presented data of our study on liver transplantation in CRGN patients nevertheless underscores the importance of a high level of alertness and the need for an interdisciplinary approach including specialists in infectiology to address CRGN infections in patients undergoing LT.

While our study indicates that the incidence of MDRO colonization rises during waiting time for LT and that colonization itself is associated with increased mortality, some limitations have to be taken into account when interpreting the data. First, MDRO colonization may represent a surrogate parameter since they are more often detected in sicker patients; however, our data show that MELD score was equal in MDRO-positive patients at LT. Second, data analysis was done retrospectively, and records of infectious complications were not prospectively defined per protocol. Third, as a consequence of the longitudinal study design, the mean survival time could not be calculated reliably. Fourth, epidemiological data of this single-center study may vary from others since local microbiological patterns differ significantly in between regions worldwide [7].

On the other hand, some important strengths of our study may be highlighted. The infection control strategy of our center ensured a literally complete screening of LT candidates at evaluation and consistent MDRO surveillance thereafter. Moreover, extending over a period of nine years, this study covers one of the longest time spans in the field of MDRO bearers on the LT waiting list. Finally, prevalence and incidence data are reliable due to the structured MDRO infection control protocols at our center and our cohort appropriately reflects a real-world scenario in a high-MELD era. Thus, concise MDRO screening and surveillance seem essential in LT candidates. Moreover, any extension of waiting time puts LT candidates on risk for MDRO colonization and subsequently increased mortality.

In conclusion, MDRO colonization is common and an independent predictive factor for mortality in LT candidates. The occurrence of CRGN resembles a major event in these patients and must be addressed with particular concern, possibly warranting infectiologic stewardship. Moreover, our findings emphasize the need for strategies to reduce the waiting time for LT and overcome organ shortage. Future research should aim at decolonization strategies as well as disrupting the progression from MDRO colonization to infection in these patients.

## Supporting information

S1 Table Etiology of liver disease in 351 patients. Liver cirrhosis was due to multiple causes in 30 patients, resulting in 381 observations altogether. Abbreviations: HBV/HDC, viral hepatitis B and D coinfection. HBV, viral hepatitis B. PSC, primary sclerosing cholangitis. SSC, secondary sclerosing cholangitis. PBC, primary biliary sclerosis. AIH, autoimmune hepatitis. ADPKD, autosomal dominant polycystic kidney disease. ALF, acute liver failure.
(DOCX)

S2 Table Number of patients repetitively screened for MDRO before undergoing LT. Among 351 patients who were screened for any MDRO at LT listing, 261 underwent at least one further MDRO screening series.
(DOCX)

S3 Table Localization of MDRGN colonization detected in 89 patients by screening smear swabs throughout the study, including patients within the entire cohort and after LT, and including evidence of second and third MDRGN. Percentages of screening results are calculated in relation to the respective number of individual strains detected throughout the study (n = 104) and after LT (n = 30), since these pathogens often have been detected repetitively. CRGN are a MDRGN subgroup that are resistant against carbapenems beside ESBL phenotype (*Enterobacterales*) or resistance against piperacillin, cefatizidim and fluoroquinolones (*P*. *aeruginosa*).
(DOCX)

S4 Table Localizations of MDRGN samples obtained in patients with clinically suspected infections within the entire cohort (n = 78 invasive detections) and after LT (n = 34 invasive detections). Percentages of test results are calculated in relation to the total number of individual positive tests, since these pathogens often have been detected in multiple body compartments.
(DOCX)

S5 Table Gram-positive colonization detected by screening smear swabs throughout the study, including patients within the entire cohort and after LT. Percentages of screening results are calculated in relation to the respective number of individual strains detected, since these pathogens often have been detected repetitively.
(DOCX)

S6 Table Localizations of Gram-positive samples obtained in patients with clinically suspected infections within the entire cohort and after LT). Percentages of test results are calculated in relation to the total number of individual positive tests, since these pathogens often have been detected in multiple body compartments.
(DOCX)

S7 Table Strains and resistance patterns in 89 patients with MDRGN positivity. Abbreviations: ESBL, extended-spectrum beta-lactamase; QR, quinolone resistance. CRGN are a MDRGN subgroup that are resistant against carbapenems beside ESBL phenotype (*Enterobacterales*) or resistance against piperacillin, ceftazidim and fluoroquinolones (*P*. *aeruginosa*).
(DOCX)

## Abbreviations

CI: confidence interval
CR: carbapenem resistance
CRGN: carbapenem-resistant gram-negative bacteria
ESBL: extended spectrum beta-lactamase
IQR: interquartile range
LT: liver transplantation
MDRGN: multidrug-resistant gram-negative bacteria
MDRO: multidrug-resistant organisms
MELD: model for end-stage liver disease
MiQ: German microbiological procedure quality standards
MRSA: methicillin-resistant *Staphylococcus aureus*
QR: fluoroquinolone resistance
VRE: vancomycin-resistant enterococci
