# Supplementary material for: Colonization with multidrug-resistant organisms is associated with in increased mortality in liver transplant candidates
Source: PLoS One. 2021 Jan 22;16(1):e0245091. doi: 10.1371/journal.pone.0245091 (PMC7822319; doi:10.1371/journal.pone.0245091)
Supplement: S1 Table — Liver cirrhosis was due to multiple causes in 30 patients, resulting in 381 observations altogether. Abbreviations: HBV/HDC, viral hepatitis B and D coinfection. HBV, viral hepatitis B. PSC, primary sclerosing cholangitis. SSC, secondary sclerosing cholangitis. PBC, primary biliary sclerosis. AIH, autoimmune hepatitis. ADPKD, autosomal dominant polycystic kidney disease. ALF, acute liver failure. (DOCX) [file pone.0245091.s001.docx]

| **Etiology** | | **n (%)** |
| --- | --- | --- |
| Ethyltoxic | | 109 (31%) |
| HCV | | 103 (29.3%) |
| Cryptogenous | | 27 (7.7%) |
| HBV/ HDV | | 21 (6%) |
| HBV | | 20 (5.7%) |
| NASH | | 20 (5.7%) |
| PSC | | 17 (4.8%) |
| SSC | | 7 (2%) |
| PBC | | 5 (1.4%) |
| Overlap AIH/PSC | | 5 (1.4%) |
| ADPKD | | 4 (1.1%) |
| Drug-induced | | 4 (1.1%) |
| AIH | | 4 (1.1%) |
| Transplant failure | | 3 (0.9%) |
| Hemochromatosis | | 2 (0.6%) |
| M. Wilson | | 2 (0.6%) |
| Miscellaneous | | 11 (3.1%) |
| Thereof with cirrhosis due to multiple etiologies | 30 (8.6%) |  |
| ALF | | 17 (4.8%) |

**Table S1: Etiology of liver disease in 351 patients.** Liver cirrhosis was due to multiple causes in 30 patients, resulting in 381 observations altogether. Abbreviations: HBV/HDC, viral hepatitis B and D coinfection. HBV, viral hepatitis B. PSC, primary sclerosing cholangitis. SSC, secondary sclerosing cholangitis. PBC, primary biliary sclerosis. AIH, autoimmune hepatitis. ADPKD, autosomal dominant polycystic kidney disease. ALF, acute liver failure.
