# Supplementary material for: Colonization with multidrug-resistant organisms is associated with in increased mortality in liver transplant candidates
Source: PLoS One. 2021 Jan 22;16(1):e0245091. doi: 10.1371/journal.pone.0245091 (PMC7822319; doi:10.1371/journal.pone.0245091)
Supplement: S2 Table — Among 351 patients who were screened for any MDRO at LT listing, 261 underwent at least one further MDRO screening series. (DOCX) [file pone.0245091.s002.docx]

| **Additional MDRO colonization screenings on the waiting list** | **261 patients (74.3%)** |
| --- | --- |
| 1 | 70 (19.9%) |
| 2 | 60 (17.1%) |
| 3 | 38 (10.8%) |
| 4 | 19 (5.4%) |
| 5 | 19 (5.4%) |
| 6 | 16 (4.6%) |
| 7 | 12 (3.4%) |
| 8 | 5 (1.4%) |
| 9 | 6 (1.7%) |
| 10 or more | 16 (4.6%) |
|  |  |

**Table S2: Number of patients repetitively screened for MDRO before undergoing LT.** Among 351 patients who were screened for any MDRO at LT listing, 261 underwent at least one further MDRO screening series.
