# Supplementary material for: Colonization with multidrug-resistant organisms is associated with in increased mortality in liver transplant candidates
Source: PLoS One. 2021 Jan 22;16(1):e0245091. doi: 10.1371/journal.pone.0245091 (PMC7822319; doi:10.1371/journal.pone.0245091)
Supplement: S3 Table — Percentages of screening results are calculated in relation to the respective number of individual strains detected throughout the study (n = 104) and after LT (n = 30), since these pathogens often have been detected repetitively. CRGN are a MDRGN subgroup that are resistant against carbapenems beside ESBL phenotype (Enterobacterales) or resistance against piperacillin, cefatizidim and fluoroquinolones (P. aeruginosa). (DOCX) [file pone.0245091.s003.docx]

| **Compartment of MDRGN detection** | **ESBL** | **ESBL + QR** | **CRGN** | **∑ MDRGN** |
| --- | --- | --- | --- | --- |
| Rectal screening | 28 (96.6%) | 51 (82.3%) | 10 (71.4%) | 89 (84.8%) |
| *Thereof after LT* | *4 (80%)* | *17 (77.3%)* | *3 (75%)* | *24 (80%)* |
| Cutaneous screening | 2 (6.9%) | 7 (11.3%) | 0 | 9 (8.6%) |
| *Thereof after LT* | *0* | *1 (4.5%)* | *0* | *1 (3.3%)* |
| Pharyngeal screening | 3 (10.3%) | 8 (12.9%) | 4 (28.6%) | 15 (14.3%) |
| *Thereof after LT* | *2 (40%)* | *2 (9%)* | *1 (25%)* | *5 (16.7%)* |

**S3 Table: Localization of MDRGN colonization detected in 89 patients by screening smear swabs throughout the study, including patients within the entire cohort and after LT, and including** **evidence of second and third MDRGN.** Percentages of screening results are calculated in relation to the respective number of individual strains detected throughout the study (n=104) and after LT (n=30), since these pathogens often have been detected repetitively. CRGN are a MDRGN subgroup that are resistant against carbapenems beside ESBL phenotype (*Enterobacterales*) or resistance against piperacillin, cefatizidim and fluoroquinolones (*P. aeruginosa*).
