# Supplementary material for: Colonization with multidrug-resistant organisms is associated with in increased mortality in liver transplant candidates
Source: PLoS One. 2021 Jan 22;16(1):e0245091. doi: 10.1371/journal.pone.0245091 (PMC7822319; doi:10.1371/journal.pone.0245091)
Supplement: S4 Table — Percentages of test results are calculated in relation to the total number of individual positive tests, since these pathogens often have been detected in multiple body compartments. (DOCX) [file pone.0245091.s004.docx]

| **Compartment of MDRGN**  **detection** | **ESBL** | **ESBL + QR** | **CRGN** | **∑ MDRGN** |
| --- | --- | --- | --- | --- |
| Ascites | 3 (30%) | 5 (11.4%) | 2 (8.3%) | 10 (12.8%) |
| *Thereof after LT* | *1 (25%)* | *1 (4.4%)* | *0* | *2 (5.9%)* |
| Urine | 1 (10%) | 18 (40.1%) | 6 (25%) | 25 (23.4%) |
| *Thereof after LT* | *1 (25%)* | *9 (39.1%)* | *1 (16.7%)* | *11 (32.4%)* |
| Blood | 2 (20%) | 5 (11.4%) | 1 (4.2%) | 8 (7.6%) |
| *Thereof after LT* | *0* | *1 (4.4%)* | *0* | *1 (2.9%)* |
| Other body fluids | 3 (30%) | 10 (22.7%) | 8 (33.3%) | 21 (26.9%) |
| *Thereof after LT* | *2 (50%)* | *7 (30.4%)* | *3 (50%)* | *13 (38.2%)* |
| Wound/surgical site | 1 (10%) | 3 (6.8%) | 4 (16.7%) | 8 (10.3%) |
| *Thereof after LT* | *0* | *3 (13%)* | *2 (33.3%)* | *5 (14.7%)* |
| Devices | 0 | 3 (6.8%) | 3 (12.5%) | 6 (7.7%) |
| *Thereof after LT* | *0* | *2 (8.7%)* | *0* | *2 (2.6%)* |
| Total invasive detections | 10 (100%) | 44 (100%) | 24 (100%) | 78 (100%) |
| *Thereof after LT* | *4 (100%)* | *23 (100%)* | *6 (100%)* | *34 (100%)* |

**S4 Table: Localizations of MDRGN samples obtained in patients with clinically suspected infections within the entire cohort (n=78 invasive detections) and after LT** **(n=34 invasive detections).** Percentages of test results are calculated in relation to the total number of individual positive tests, since these pathogens often have been detected in multiple body compartments.
