# Supplementary material for: Colonization with multidrug-resistant organisms is associated with in increased mortality in liver transplant candidates
Source: PLoS One. 2021 Jan 22;16(1):e0245091. doi: 10.1371/journal.pone.0245091 (PMC7822319; doi:10.1371/journal.pone.0245091)
Supplement: S5 Table — Percentages of screening results are calculated in relation to the respective number of individual strains detected, since these pathogens often have been detected repetitively. (DOCX) [file pone.0245091.s005.docx]

|  |  |  |  |  |
| --- | --- | --- | --- | --- |
|  | **∑ MRSA** | **∑ VRE** | **Thereof teicoplanin- resistant** | **Thereof linezolid- or tigecycline- resistant)** |
| Rectal | 3 (15.8%) | 111 (90.2%) | 19 (95%) | 8 (100%) |
| *Thereof after LT* |  | *21 (87.5%)* | *6 (30%)* | *2 (100%)* |
| Cutaneous | 3 (15.8%) | 7 (5.7%) | 1 (5%) |  |
| *Thereof after LT* |  | *1 (4.2%)* |  |  |
| Pharyngeal/throat | 17 (89.5%) | 4 (3.3%) |  |  |
| *Thereof after LT* |  | *1 (4.2%)* |  |  |
| **Patients** | 19 (100%) | 123 (100%) | 20 (100%) | 8 (100%) |
| *Thereof after LT* |  | *24 (100%)* | *7 (100%)* | *2 (100%)* |

**S5 Table: Gram-positive colonization detected by screening smear swabs throughout the study, including patients within the entire cohort and after LT.** Percentages of screening results are calculated in relation to the respective number of individual strains detected, since these pathogens often have been detected repetitively.
