# Supplementary material for: Colonization with multidrug-resistant organisms is associated with in increased mortality in liver transplant candidates
Source: PLoS One. 2021 Jan 22;16(1):e0245091. doi: 10.1371/journal.pone.0245091 (PMC7822319; doi:10.1371/journal.pone.0245091)
Supplement: S6 Table — Percentages of test results are calculated in relation to the total number of individual positive tests, since these pathogens often have been detected in multiple body compartments. (DOCX) [file pone.0245091.s006.docx]

|  | **∑ MRSA** | **∑ VRE** | **Thereof teicoplanin- resistant** | **Thereof linezolid- or tigecycline- resistant)** |
| --- | --- | --- | --- | --- |
| Ascites | 1 (5.3%) | 10 (8.5%) | 1 (12.5%) | 2 (18.2%) |
| *Thereof after LT* | *0* | *0* | *0* | *0* |
| Urine | 2 (10.5%) | 21 (17.8%) |  | 2 (18.2%) |
| *Thereof after LT* |  | *4 (9.5%)* |  | *1 (14.3%)* |
| Blood | 0 | 17 (14.4%) | 2 (25%) | 2 (18.2%) |
| *Thereof after LT* |  | *7 (16.7%)* | *2 (33%)* | *1 (14.3%)* |
| Other body fluids | 2 (10.5%) | 30 (25.4%) | 3 (37.5%) | 2 (18.2%) |
| *Thereof after LT* |  | *10 (23.8%)* | *2 (33%)* | *4 (57.1%)* |
| Wound | 1 (5.3%) | 23 (19.5%) | 2 (25%) | 3 (27.3%) |
| *Thereof after LT* |  | *15 (35.7%)* | *2 (33%)* | *1 (14.3%)* |
| Devices |  | 17 (14.4%) |  |  |
| *Thereof after LT* |  | *6 (14.3%)* | *0* | *0* |
| Total invasive detections | 6 (100%) | 118 (100%) | 8 (100%) | 11 |
| *Thereof after LT* | *0 (100%)* | *42 (100%)* | *6 (100%)* | *7 (100%)* |

**S6 Table: Localizations of Gram-positive samples obtained in patients with clinically suspected infections within the entire cohort and after LT.** Percentages of test results are calculated in relation to the total number of individual positive tests, since these pathogens often have been detected in multiple body compartments.
