# Supplementary material for: Colonization with multidrug-resistant organisms is associated with in increased mortality in liver transplant candidates
Source: PLoS One. 2021 Jan 22;16(1):e0245091. doi: 10.1371/journal.pone.0245091 (PMC7822319; doi:10.1371/journal.pone.0245091)
Supplement: S7 Table — Abbreviations: ESBL, extended-spectrum beta-lactamase; QR, quinolone resistance. CRGN are a MDRGN subgroup that are resistant against carbapenems beside ESBL phenotype (Enterobacterales) or resistance against piperacillin, ceftazidim and fluoroquinolones (P. aeruginosa). (DOCX) [file pone.0245091.s007.docx]

|  |  |  |  |  |
| --- | --- | --- | --- | --- |
|  | **ESBL** | **ESBL + QR** | **CRGN** | **∑ MDRGN** |
|  |  |  |  |  |
| *C. freundii* |  | 1 |  | 1 |
| *C. koseri* |  | 1 |  | 1 |
| *E. aerogenes* |  | 1 |  | 1 |
| *E. cloacae* | 1 | 4 |  | 5 |
| *Thereof after LT* |  | *2* |  | *2* |
| *E. coli* | 15 | 39 |  | 54 |
| *Thereof after LT* | *4* | *3* |  | *7* |
| *K. pneumoniae* | 13 | 15 | 7 | 35 |
| *Thereof after LT* | *5* | *10* | *3* | *18* |
| *P. aeruginosa* |  |  | 7 | 7 |
| *Thereof after LT* |  |  | *2* | *3* |
| *Total individual strains* | 29 | 62 | 14 | 104 |
| *Thereof after LT* | *9* | *16* | *5* | *30* |

**S7 Table: Strains and resistance patterns in 89 patients with MDRGN positivity.** Abbreviations: ESBL, extended-spectrum beta-lactamase; QR, quinolone resistance. CRGN are a MDRGN subgroup that are resistant against carbapenems beside ESBL phenotype (*Enterobacterales*) or resistance against piperacillin, ceftazidim and fluoroquinolones (*P. aeruginosa*).
